# Adsorption of Phenylalanine from Aqueous Solutions Using Activated Carbon from Sunflower Meal Functionalized with Sulfonic Groups

**DOI:** 10.3390/foods11213427

**Published:** 2022-10-29

**Authors:** William Cardoso Lima, Leandro S. Oliveira, Adriana S. Franca

**Affiliations:** 1 Graduate Program in Food Science, Faculdade de Farmácia, Universidade Federal de Minas Gerais, Av. Antônio Carlos, 6627, Belo Horizonte 31270-901, Brazil; 2 Federal Institution of Education, Science and Technology of Mato Grosso, MT-235, Km 12-Zona Rural, Campo Novo do Parecis 78360-000, Brazil; 3 Mechanical Engineering Department, Universidade Federal de Minas Gerais, Av. Antônio Carlos, 6627, Belo Horizonte 31270-901, Brazil

**Keywords:** agro-food waste, sulfanilic acid, amino acid, sulfonation, phenylketonuria

## Abstract

The present work proposes the use of an agro-industrial residue from the sunflower crop as a feedstock to produce a low-cost adsorbent with a chemically modified surface bearing sulfonic groups. This modified low-cost adsorbent can be used for the removal of phenylalanine, and can also be applied in the process of obtaining a source of protein supplementation for patients with phenylketonuria. The functionalization of the adsorbent with sulfonic groups was adapted and presented advantages in terms of execution time, energy expenditure, number of reagents used and adsorbed amino acids. The produced adsorbent presented a surface area of 317.31 m^2^ g^−1^ with a predominance of micro- and mesopores, that influenced an approximate 30-fold reduction in adsorption equilibrium time. The optimization results indicated a higher adsorption capacity (39.64 mg g^−1^) in pH = 4; temperature of 25 °C and adsorbent dosage of 10 g L^−1^. The FTIR analyzes and the qualitative analysis of the elements present in the samples by EDS confirmed the introduction of sulfonic groups in the MPS500 coal. This work contributed to the understanding behind the adsorption of L-phenylalanine on charcoal surfaces functionalized with sulfonic groups, showing that they can be more selective for the adsorption of phenylalanine in a competitive system.

## 1. Introduction

Phenylalanine is an essential amino acid with a non-polar characteristic due to the presence of an aromatic ring in its structure. Its consumption should be avoided by individuals with phenylketonuria (PKU), which is a metabolic disorder characterized by a deficiency of the enzyme phenylalanine hydroxylase that catalyzes the hydroxylation of phenylalanine to tyrosine [1]. Excess phenylalanine can lead to the development of postnatal cognitive impairment, epileptic seizures, microcephaly, growth failure and poor skin pigmentation, if not diagnosed and treated with a strict diet [2]. The diet implements a restriction on protein sources, leading to low phenylalanine consumption, and must be maintained for life; thus, individuals with PKU need to consume protein substitutes as a complementary source of amino acids [3].

The organoleptic properties of protein substitutes are undesirable to consumers due to poor palatability and breath odor, causing inconvenience to patients with PKU [3]. Furthermore, regardless of the type of protein substitute and phenylalanine-free formulations, the costs incurred to patients in treatment are high [4]. One way to reduce costs and produce more palatable formulations is the use of protein hydrolysates with acceptably low phenylalanine content. Studies have shown that whey, milk, rice, wheat flour and egg white protein hydrolysates with low phenylalanine content are promising alternatives to costly protein substitutes [5,6,7,8,9,10]. However, the removal of this amino acid is necessary to attain the low concentrations required for PKU patients [11]. The removal of phenylalanine from protein hydrolysates is usually carried out by adsorption of the target amino acid on adsorbents such as activated carbons and polymeric resins [5,8,9,12,13].

In the agri-food sector, activated carbon (AC) can be applied in the decolorization of vegetable oils and sugars, removal of copper ions and undesirable organic compounds present in distilled alcoholic beverages, in the removal of phenylalanine from protein hydrolysates and other applications [4]. Despite all the applications mentioned, the use of activated carbon presents certain drawbacks related to the high costs of the precursor materials, energy expenditure involved in its preparation [4], non-selectivity towards a specific adsorbate, and the costly regeneration process of the saturated activated carbon [14], all aspects that increase the value of industrialized products produced thereof. To tackle these issues, agri-food wastes have been extensively studied as low-cost precursor materials to produce activated carbons, employing as little energy as possible [15]. Chemical functionalization of activated carbon is usually employed to improve selectivity towards a specific target adsorbate [16].

Agricultural wastes have been successfully studied as precursor materials for the production and utilization of activated carbons for the removal of phenylalanine from aqueous solutions [13,17,18]. Clark et al. [13] prepared an adsorbent by activating a phosphoric-acid-treated defective coffee press cake at 350 °C. Batch adsorption studies were conducted and the determined maximum uptake capacity of phenylalanine was comparable to those published in the literature for synthetic adsorbents. Hydrophobic interactions of the π–π type, between carbon rings at the adsorbent surface and phenyl rings of the adsorbate molecules, were determined to be the predominant adsorption mechanism. In a subsequent study, Alves et al. [17] prepared an activated carbon-based adsorbent utilizing corn cobs as precursor material and used the adsorbent to remove tyrosine and phenylalanine from aqueous solutions. Both single component and binary solutions were studied. Although the adsorption behavior of tyrosine in single component solution was similar to that of phenylalanine, the respective adsorption capacity was significantly lower than that of phenylalanine. The studies with binary solutions demonstrated that phenylalanine will be preferentially adsorbed compared to tyrosine. The occurrence of distinct adsorption mechanisms were demonstrated in the case of phenylalanine single solution with hydrophobic π–π interactions being considered the primary one. Other types of interactions were hydrogen bonding between the oxygenated acidic groups at the adsorbent surface and the amino group of the phenylalanine molecule, imido-type bonds between the phenylalanine amino groups and the carboxyl groups at the adsorbent surface, and formation of phosphonates via the interaction of phenylalanine carboxylic groups with phosphate groups linking graphene sheets of the adsorbent main structure.

Belhamdi et al. [18] prepared two distinct activated carbons by individually treating date stones with potassium hydroxide and with zinc chloride and used the prepared carbons as adsorbents for the removal of phenylalanine from aqueous solutions. The AC prepared with potassium hydroxide presented better adsorption performance than the one prepared with zinc chloride. Phenylalanine adsorption onto the carbon surface was demonstrated to be strongly dependent on the solution pH, with the highest adsorption capacity occurring at a pH corresponding to the isoelectric point of the phenylalanine molecule (pH 5.7). The predominant adsorption mechanism was deemed to be of the π–π type with other minor mechanisms being of the hydrogen bonding type between the amino groups of phenylalanine and the oxygenated groups at the carbon surface.

In view of the aforementioned, the present work proposes the use of sunflower seed meal, a by-product from the extraction of sunflower seed oil, as low-cost precursor material for the preparation of sulfonated activated carbon to be used for the removal of phenylalanine from aqueous solutions.

## 2. Materials and Methods

### 2.1. Material

The sunflower meal used in this work was donated by Indústria Parecis SA (Campo Novo do Parecis, MT, Brazil). Phosphoric acid (85% *w*/*w*), hydrochloric acid and sodium hydroxide were purchased from VETEC (Rio de Janeiro, RJ, Brazil), sulfanilic acid and sodium nitrite were purchased from Neon (Suzano, SP, Brazil).

### 2.2. Methods

#### 2.2.1. Activated Carbon Preparation

Sunflower meal was chemically activated by impregnation with phosphoric acid (85%) in a 1:1 ratio (*w*/*v*), stirred for 3 min at room temperature, and carbonized in a conventional muffle oven (EDG, EDG3P-S model, São Carlos, SP, Brazil) according to the methodology of Zabaniotou et al. [19] with modifications. Sunflower meal (7 g) was placed in semi-closed porcelain crucibles and heated from room temperature to 500 °C, with a heating rate of 10 °C min^−1^, and a residence time of 10 min without controlled atmosphere. The obtained char was washed with enough distilled water to remove organic and mineral matter residues, and dried in a convective oven (Nova Etica, model 420, São Paulo, SP, Brazil) set at 105 °C for 4 h. The samples were then identified as M500 (without chemical activation) and MP500 (activation with phosphoric acid).

Functionalization of the prepared activated carbons with sulfonic groups was performed combining the published methodologies by Konwar et al. [20], Malins et al. [21] and Shengli et al. [22]. In total, 5.2 g of sulfanilic acid and 3 g of MP500 were added in an Erlenmeyer flask containing 300 mL of 1 mol L^−1^ aqueous hydrochloric acid solution, the mix was kept under constant stirring. A dropwise addition of 33 mL of sodium nitrite solution (1 mol L^−1^) was then performed for a maximum period of 5 min. The reaction took place for another 15 min in an incubator with orbital shaking at 200 rpm and a controlled temperature of 45 °C. The functionalized activated carbon was vacuum filtered and washed with water until attaining a neutral pH. Then, the final samples were dried in a convective oven at 105 °C for 4 h and the resultant product was identified as MPS500.

The gravimetric yield of the obtained ACs from each step was calculated by

%Gravimetric yield = (activated carbon mass)/(sunflower meal mass) × 100(1)

#### 2.2.2. Characterization of the Adsorbents

Thermal analyses of the samples were performed in a TGA-51 thermogravimetric analyzer (Shimadzu). Approximately 20 mg of each sample was placed in platinum crucibles, and nitrogen (99.9% purity) was used as carrier gas, with a flow rate of 120 mL min^−1^. The temperature ranged from 25 to 900 °C, with a heating rate of 10 °C min^−1^. 

Specific surface area and pore volume were determined with nitrogen adsorption and desorption isotherms at −196 °C (Quantachrome, Nova 1200e model, EUA). The specific surface area was calculated by the Brunauer–Emmett–Teller (BET) method. The pore size and the total volume were calculated by the Barrett–Joyner–Halenda equation, while the micropore volume was calculated by the t method [18].

Fourier Transform Infrared Spectroscopy (FTIR) analyses of the activated carbon samples were performed in an IRAFFINITY-1 Spectrophotometer (Shimadzu), in the range of 4000 to 400 cm^−1^, and a resolution of 4 cm^−1^. The spectrum of the sulfonated carbon was compared with the chemical groups determined by the Boehm method, according to the methodology described by Clark et al. [13]. The number of sulfonic acid sites was quantified according to the methodology proposed by Nata et al. [23]. The point of zero charge of the sulfonated adsorbent (pH_PZC_) was determined according to the method described by Clark et al. [13].

The morphologies of the AC samples were analyzed in a scanning electron microscope, coupled with an energy dispersion X-ray spectroscopy (Hitachi, model TM3000). The samples were placed on carbon strips and analyzed at 500-, 1000- and 2000-times magnification. Qualitative analyses of the elements present in the samples by EDS were performed in four randomly chosen regions.

#### 2.2.3. Adsorption Test

The sulfonated ACs were tested for their adsorption capacity, according to the methodology described by Alves et al. [17], with modifications. The effects of varying parameters, such as particle size (1.0–0.75 mm), AC dosage (5–40 g L^−1^), initial pH of the solution (2–8), temperature (25–45 °C) and initial concentration of phenylalanine (100–500 mg L^−1^) were evaluated. The sulfonated ACs were added to Erlenmeyer flasks containing 150 mL of phenylalanine solutions of known concentration, which were placed in an incubator with orbital shaking (Quimis, model Q816M28, Diadema, SP, Brazil) at 100 rpm. An aliquot of the solution was collected from the flasks at intervals of 25 min and the absorbance was measured in a UV–Vis spectrophotometer (Spectrumlab, Gold model S53, Shanghai, China) a wavelength of 257 nm, characteristic of phenylalanine. The concentration of the aliquot was calculated using calibration curves. The adsorbed amount per gram of adsorbent (qt) as a function of time was determined by

q_t_ = (C_i_ − C_t_) V/m (2)

where C_i_ and C_t_ are the concentrations of the adsorbate at the initial time and at a time t in the solution (mg L^−1^), V is the volume of the solution (L) and m is the mass of adsorbent used (g). The pH of the phenylalanine solution before and after the adsorption process was measured using a microprocessor pH meter (Quimis brand, model Q400MT, Diadema, SP, Brazil).

The efficiency of the prepared adsorbent (I) was calculated based on the evaluation of the adsorption capacity (q_t_) and the gravimetric yield (R) as:

I = q_t_ × R (3)

The total removal efficiency (R%) was calculated according to Equation (4):

R = ((C_o_ − C_e_)/C_o_) × 100 (4)

where C_o_ is the initial concentration of L–phenylalanine and C_e_ is the equilibrium concentration of L–phenylalanine [24].

#### 2.2.4. Adsorption Tests with Binary Solutions Containing Phenylalanine and Tyrosine

The evaluation of binary systems was performed according to the methodology proposed by Alves et al. [25]. Adsorption tests using Tyrosine monocomponent solution with concentrations of 20, 30, 50, 75 and 100 mg L^−1^ were carried out. For the adsorption tests using binary solutions, the concentration of the interfering amino acid was fixed and the concentration of the primary amino acid was varied as follows: (a) Phe as primary amino acid with a concentration ranging from 100 to 500 mg L^−1^ and Tyr as an interferent with a concentration of 50 mg L^−1^; (b) Tyr as the primary amino acid with a concentration ranging from 20 to 100 mg L^−1^ and Phe as an interferent with a concentration of 500 mg L^−1^. All solutions included in the adsorption tests were used with pH = 4, temperature of 25 °C, and carbon dosage of 10 g L^−1^. An aliquot of the solution was collected at intervals of 25 min, and the absorbance was measured in a UV–Vis spectrophotometer (Spectrumlab, Gold model S53, Shangai, China) using a wavelength of 257 nm and 273 nm, for phenylalanine and tyrosine, respectively. The concentration of the aliquot was calculated using calibration curves. The adsorbed amount per gram of adsorbent (qt) as a function of time was determined by Equation (2) (Section 2.2.3).

#### 2.2.5. Statistical Analysis

All the analyses were performed in triplicates. The results were submitted to a Tukey test at 5% significance, and to an analysis of variance (ANOVA) (Minitab Professional software version 16.1).

## 3. Results and Discussion

### 3.1. Adsorbent Preparation

The data for gravimetric yield (%), adsorption capacity (q_t_), performance index (I), and total removal efficiency (R%) for samples M500, MP500 and MPS500 are presented in Table 1.

MP500 and MPS500 samples presented higher gravimetric yields than M500 AC (Table 1). This result can be explained by the chemisorption of oxygen in thermal treatment without atmospheric control, and by the high ratio of phosphoric acid impregnation [26].

Moralı et al. [27] also used sunflower meal to produce activated carbon in a conventional oven (muffle) using the same temperature. However, in their work, the atmosphere and methodology used for impregnation with phosphoric acid differed from those used in the present study, resulting in gravimetric yields two- to four-times lower than those of the MP500 and MPS500 ACs prepared herein (Table 1).

Activation with phosphoric acid demonstrated a positive effect by increasing the phenylalanine adsorption capacity of MP500 when compared to that of M500 (Table 1). Chemical modification of the carbon surface with the introduction of sulfonic groups increased the adsorption capacity of phenylalanine by approximately 47%, when MPS500 sample was compared with MP500, and by approximately 254% when compared with sample M500 (Table 1), in addition to increasing the total phenylalanine removal efficiency by approximately four times when compared to M500 charcoal. MPS500 presented a lower gravimetric yield than MP500. However, the adsorption capacity of the sulphonated sample was higher. It was observed that the MPS500 sample presented a performance index five-times greater than the M500 sample, and the MP500 sample resulted in an efficiency four-times higher than the M500 sample.

### 3.2. Adsorbent Characterization

**Figure 1:**
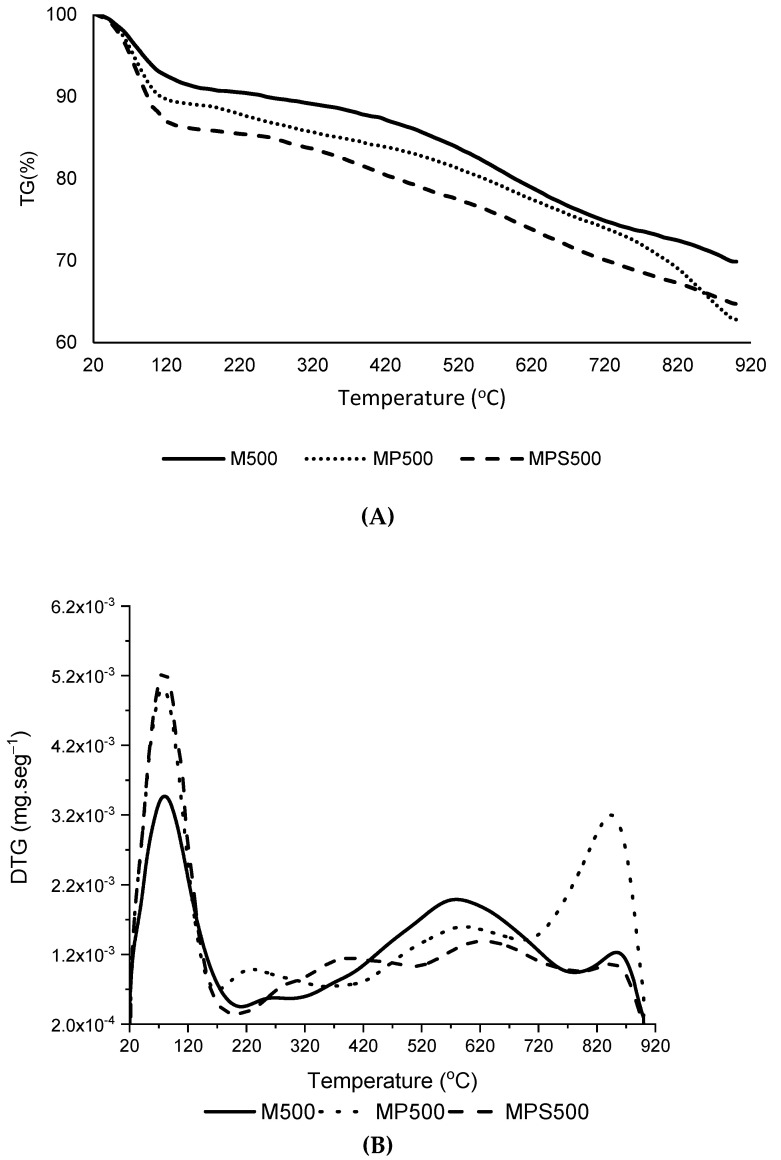
TG curves (**A**) and DTG curves (**B**) in inert atmosphere of the control AC (M500), AC treated with phosphoric acid (MP500) and AC treated with phosphoric acid and functionalized with sulfonic groups (MPS500).

Characterization of the adsorbent via thermogravimetric analysis (Figure 1) suggested a weight-loss up to 130 °C for all samples, which is attributed to the presence of water, both free and adsorbed on the surface of the adsorbent [28,29].

A greater amount of water was present in the MPS500 sample (13.44%) when compared to the M500 sample (7.91%). Rocha et al. [30] reported a higher moisture content in sulfonated AC than in non-functionalized AC using a methodology similar to the one herein applied. These results could be related to the functionalization of the active carbon surface with sulfonic groups, which can form hydrogen bonds with water molecules, resulting in greater moisture content in this sample when compared to M500 carbon.

Weight loss in the range of 180 to 230 °C can be attributed to the loss of oxygenated acidic groups such as hydroxyl, carboxyl and carbonyl groups [31]. In this study, it was observed that the M500 and MPS500 ACs (Figure 1B) exhibited similar behavior in this temperature range, with a mass reduction of approximately 0.5%, whereas the MP500 AC presented greater mass loss in that same region (~1.2%). The loss of mass that occurred in the temperature range of 240 to 380 °C could be attributed to the removal of—SO_3_H groups [28], and in this work MPS500 presented greater mass loss for this temperature range than the other ACs, which corroborates the effective insertion of sulfonic groups on the AC surface. The chemical functionalization of the AC promoted the formation of an adsorbent with lower thermal stability when compared to the others. This decrease in thermal stability following the introduction of sulfonic groups was also observed by Malins et al. [21], who prepared an activated carbon using a methodology similar to that used in this work.

Moreover, thermogravimetric analysis of sunflower bran (data not shown) was performed and mass reductions of 23.71% (190–320 °C), 26.93% (320–450 °C) and 15.36% (280–400 °C) were observed, referring to the release of CH_4_ and CO_2_ from the decomposition of hemicellulose, lignin and cellulose, respectively [32].

**Figure 2:**
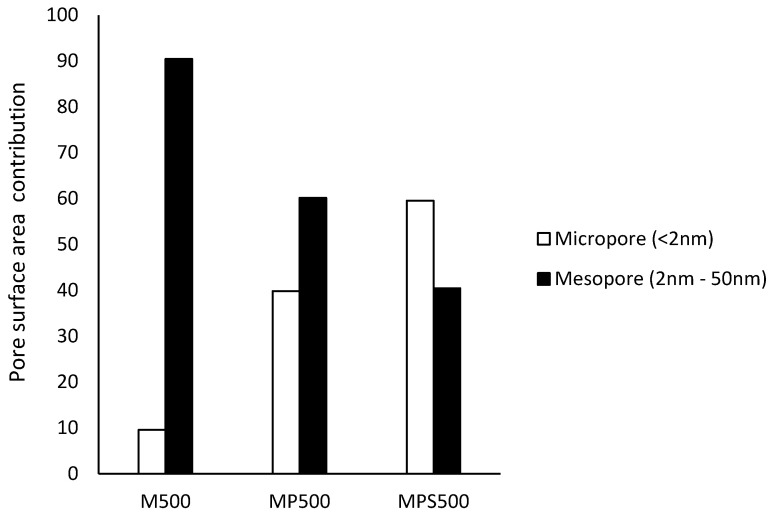
Pore distribution in M500, MP500, MPS500.

The textural properties derived from nitrogen isotherms of the prepared adsorbents showed some changes in the percentage of the composition of mesopores and micropores as a function of chemical modification performed in the adsorbents (Table 2). MPS500 presented a pore surface area of mesopores two-times smaller and pore surface area of micropores six-times larger than M500 (Figure 2).

Textural characteristics significantly influence the adsorption kinetics since the types of pores and their respective size distributions are responsible for controlling the diffusion of adsorbate into the adsorbent. Adsorbents prepared from defective coffee beans [13] and corn cobs [17] chemically activated with phosphoric acid presented lower percentage distributions of mesopores and higher amounts of micropores than those of the herein prepared adsorbents (Table 2).

These characteristics had an impact on the adsorbate diffusion process into the herein prepared adsorbent, allowing for adsorption equilibrium times approximately 29-times shorter than that of Alves et al. [17] and approximately 15-times shorter than that reported by Clark et al. [13] (Table 3), i.e., higher diffusion rates.

**Figure 3:**
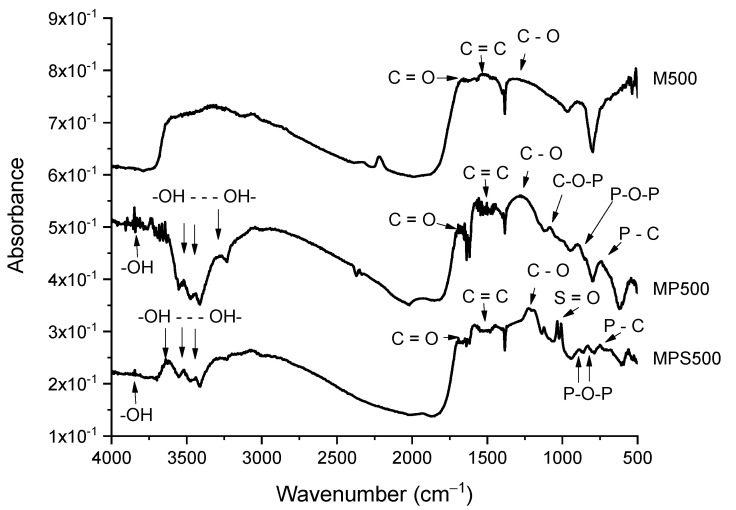
FTIR spectra of control activated carbon (M500), activated carbon treated with phosphoric acid (MP500) and activated carbon treated with phosphoric acid and further functionalized with sulfonic groups (MPS500).

The FTIR spectra of M500, MP500 and MPS500 are presented in Figure 3. The 1150–1400 cm^−1^ spectral region is associated with the CO-bond-stretching vibrations present in acids, alcohols, phenols, esters and ethers. The 1400–1600 cm^−1^ region refers to the C=C bond of aromatic compounds, and the 1650–1800 cm^−1^ region is related to C=O bond in carboxylic acid and lactone [17,18,30,33].

The MP500 and MPS500 samples showed absorption bands between 3700–3584 cm^−1^ related to stretch of the OH group in phenols, and 3550–3200 cm^−1^ related to alterations in the stretch of the OH group in phenols, associated with intermolecular hydrogen bonding. These chemical groups on the adsorbent surface have great potential for adsorption of L-phenylalanine [18,33]. In our study, we observed that these bands were present in samples with the highest adsorption capacities (MP500 and MPS500), suggesting that the presence of these hydroxyl groups may have contributed to the observed increase in adsorption capacity (Table 1). The absorption bands at the regions 1025-870 and 800–650 cm^−1^ present both in MP500 and MPS500 are, respectively, associated to the stretching of the P–O–P [34,35] and P–C bonds [17,36]. The peaks at the region 1120–1020 cm^−1^, appearing only in the MP500 sample, correspond to stretching of the C-O-P bond that is observed when esterification reactions occur between cellulose and hemicellulose chains of the carbon matrix with phosphate groups [26,28,30,37]. The AC functionalization with sulfonic groups was corroborated by the presence of a peak associated with stretching of the S=O bond in the 1051–1001 cm^−1^ region [28,29,38,39].

The oxygenated chemical groups identified in the IR spectrum of MPS500 were tentatively quantified using the Boehm method following the methodology described by Nata et al. [23]. The results showed that 91.49% of oxygenated acidic groups were distributed as sulfonic (0.43 mmol g^−1^), carboxylic (3.77 mmol g^−1^), lactonic (5.53 mmol g^−1^) and phenolic (5.54 mmol g^−1^). The predominance of acidic groups is also in accordance with the pH_PZC_ of 1.98 of this adsorbent. In addition, with the exception of the sulfonic groups, the acidic groups can be derived from the thermal degradation of precursor material in an oxidative atmosphere and from the reaction between phosphoric acid and the cellulosic and hemicellulosic fractions of the precursor material [37]. Other adsorbents prepared with phosphoric acid have also been reported in the literature with a predominance of oxygenated acidic groups at their surfaces [13,17,37].

**Figure 4:**
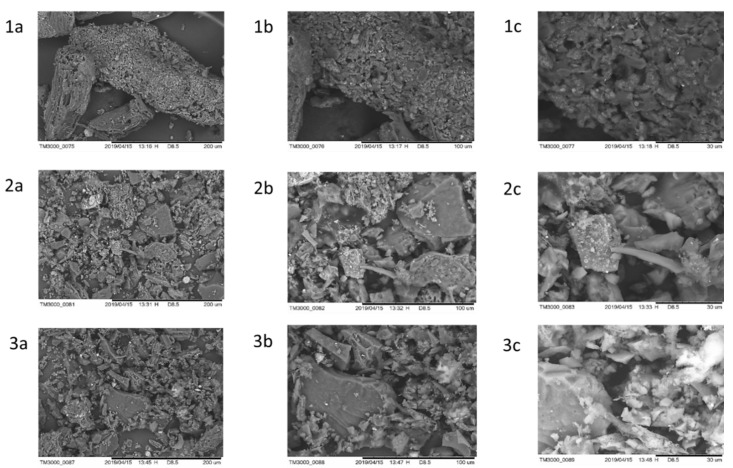
Photomicrographs of M500 (**1**), MP500 (**2**) and MPS500 (**3**) in magnifications of (**a**) 500×, (**b**) 1000× and (**c**) 2000×.

The morphologies of M500, MP500 and MPS500 were investigated via scanning electron microscopy (Figure 4).

The M500 sample images (Figure 4(1a–1c)) showed particle agglomerates with little structural diversification, and some recesses as a function of these agglomerations can also be observed (Figure 4(1c)). The MP500 and MPS500 images (Figure 4(2a–2c and 3a–3c)) exhibited more fragmented materials at the surface than the control AC (M500), indicating that the chemical treatment modified the physical structure of the AC, promoting fragmentation of the lignocellulosic material, possibly through partial acid hydrolyzation of the polysaccharides and lignin structures via the action of the phosphoric acid [40]. Morali et al. [27] observed that the interaction of phosphoric acid with sunflower meal weakened the bonds within the structure of the meal when producing activated carbon, corroborating the statement that the fragmentation observed in the present work might have occurred due to the hydrolyzing action of the activating agent. The cavities presented in the adsorbents prepared with chemical activation might have resulted from a partial evaporation of phosphoric acid during the carbonization process, emptying the space previously occupied by this chemical agent [33,41]. The MPS500 sample showed a more fragmented structure (Figure 4(3c)) when compared to the others, thus the functionalization with sulfonic groups after activation with phosphoric acid may have further weakened the bonds present in the carbon structure produced.

Energy dispersion X-ray spectroscopy analysis of samples M500, MP500 and MPS500 indicated the presence of carbon, oxygen, phosphorus, magnesium and aluminum atoms (Table 4).

Sample M500 did not present sodium and silicon atoms, while in sample MP500, sulfur and sodium atoms were not present. In the MPS500 sample, no calcium or potassium atoms were found. The increase in percentage of carbon atoms in the MPS500 sample may have occurred due to the functionalization with sulfonic groups when using sulfanilic acid, which occurred via a reaction mechanism involving the arylation of the carbon structure [42]. The amount of phosphorus increased in sample MP500 when compared to sample M500, demonstrating the success of impregnation with this acid. Interestingly, after functionalization with sulfonic groups, there was a reduction in the amount of phosphorus. This reduction may have occurred as a result of the increase in the proportion of other atoms that entered the carbon structure, or even due to the exit of phosphorus atoms in the solution used as a reaction medium.

### 3.3. Influence of Adsorbent Particle Size and Dosage and pH of the Initial Solution on the Adsorption Capacity of Phenylalanine

**Figure 5:**
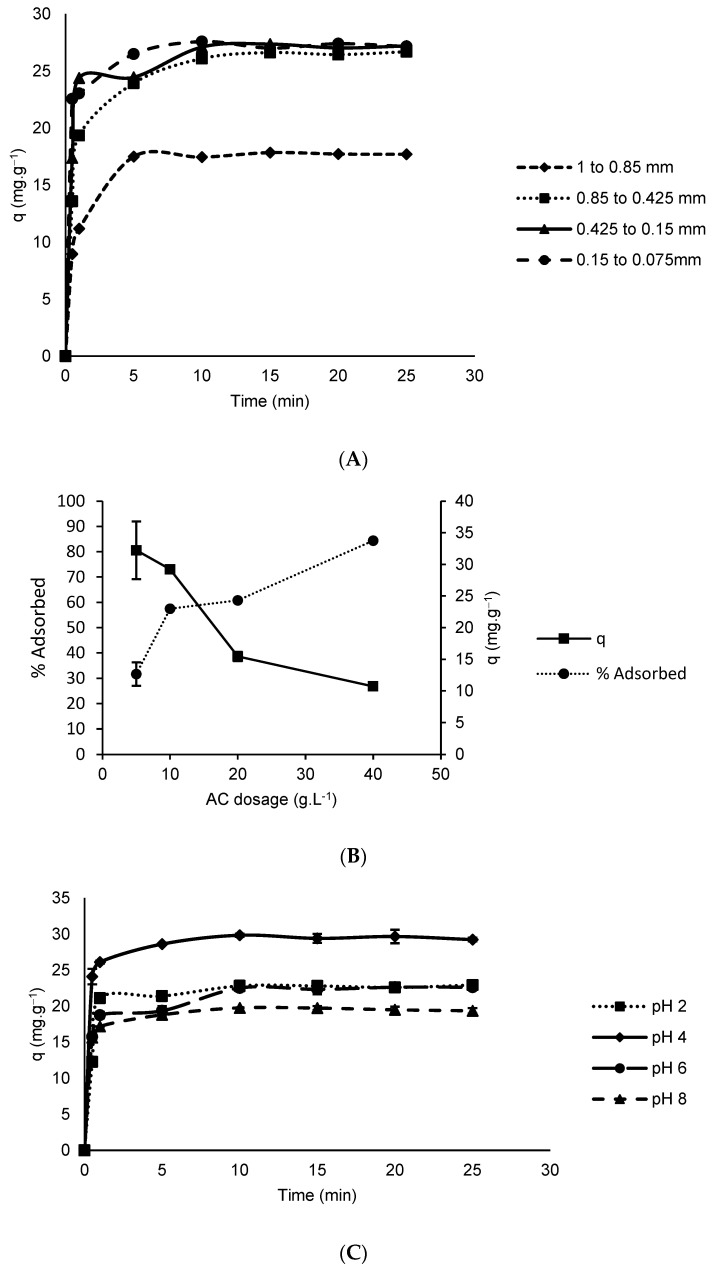
Effects of parameter variation on adsorption capacity: (**A**) adsorbent particle size; (**B**) adsorbent dosage, and (**C**) pH for phenylalanine adsorption (25 °C and initial Phe concentration of 500 mg L^−1^).

Phenylalanine adsorption increased as the particle size decreased (Figure 5A). Alves et al. [17] reported the same finding, which can be explained by the enhanced accessibility to pores in smaller-sized particles. The smallest ranges used in this work presented no statistical difference from the interval used in the continuation of the experiments, considering the yield obtained. However, in preliminary tests it was concluded that it was possible to use the adsorbent after chemical modification with sulfonic groups without performing separation of the studied distinct ranges of particle sizes (data not shown).

The variation in adsorbent dosage influenced the phenylalanine adsorption process (Figure 5B). The increase in dosage increased the percentage of removal due to a greater availability of adsorption sites. However, the adsorption capacity decreases with increasing dosage, due to a decreased adsorbate/adsorbent ratio. Based on these results, the dosage of 10 g L^−1^ was selected for further studies, as higher dosages showed a 50% reduction in adsorption capacity and at lower dosages the phenylalanine removal efficiency was unsatisfactory. Similar results were found in adsorbents produced with corn cobs and defective coffee beans [13,17].

pH is an important parameter for elucidating the adsorption mechanism of phenylalanine in aqueous solutions, which presents electrical charges that vary according to its isoelectric point and the solution pH used in the process. The phenylalanine molecule contains carboxylic and amine groups that can ionize according to the pH of the solution; therefore, electrical charges can be present in its structure. At pH values between 2.11 and 9.13, the carboxyl groups of phenylalanine molecules are deprotonated, presenting a negative charge, while amine groups are protonated with a positive charge. At pH values below 2.11, the phenylalanine molecule is positively charged with a protonated amine group, while at pH above 9.11, the molecule is negatively charged with a deprotonated carboxylic group [43]. The isoelectric point of the phenylalanine molecule is 5.48. Thus, the lower the pH value in relation to its isoelectric point, the more positively charged the molecule. In addition, the higher the pH in relation to its isoelectric point, the more negatively charged the molecule [44]. 

It is important to emphasize that pH also influences the charge presented by the adsorbent surface. Therefore, attractive or repulsive Coulomb interactions between the adsorbate and the adsorbent surface are present during solution contact, and the variation in adsorption capacity with pH can be explained by the synergism between the adsorbate–adsorbate and adsorbate–adsorbent interactions. In the pH range used in this work, the phenylalanine molecule predominantly presents a positive charge at pH 2 and pH 4, with a slight negative charge at pH 6 due to proximity to the isoelectric point pI 5.48 of this molecule [18], and a predominance of negative charge at pH 8. The adsorbent used had a negative charge in almost all the pH range studied, except for pH 2, which is equal to its pH_PZC_, at which the surface is electrically neutral, i.e., positive charges are balanced by negative charges. 

The Coulomb repulsive interactions between the adsorbate molecules varied when comparing pH 6 and pH 8. At pH 6 (Figure 5C), an increase in adsorption capacity was observed when compared to pH 8. The adsorbate–adsorbent repulsive Coulomb interactions at pH 6 were lower than at pH 8 due to the proximity of this pH to the pI of the phenylalanine molecule, consequently leading to lesser electrostatic repulsive interaction between adsorbate–adsorbent. Lower adsorption capacities at high pH values were also observed for adsorbents produced with palm seed [18], defective coffee beans [13] and corn cobs [17], when used for adsorption of phenylalanine.

At pH 4, the phenylalanine molecules are mostly positively charged whereas the adsorbent surface is negatively charged, thus favoring electrostatic attraction between the phenylalanine molecules and the surface, resulting in increased adsorption capacity at this pH value in comparison to the other pH herein studied, i.e., adsorption capacity at pH 4 was about 27, 30 and 50% higher when compared to the capacity at pH 2, 6 and 8, respectively. Some studies have also reported an adsorption favoring of phenylalanine due to electrostatic attractive interactions at pH 5.7 [18]; pH 4 and pH 6 [13,17]. At pH 2, the surface of the adsorbent presented a zero net charge, since the solution pH was equal to the pH_PZC_. Therefore, Coulomb repulsive interactions were mostly present between the adsorbate molecules, which were predominantly positively charged. At pH 8, both the adsorbent surface and the phenylalanine molecules were predominantly negatively charged and thus electrostatic repulsion between adsorbate molecules and between adsorbate and adsorbent were at its peak, incurring lower adsorption capacity at this pH when compared to the others herein studied.

Regardless of the solution pH, within the range studied, phenylalanine was adsorbed to a varied extent, suggesting that adsorption via a pH-independent mechanism had occurred. Hydrophobic interactions of the π–π type, between the aromatic rings of the adsorbent surface and the phenylalanine molecules, are independent of the solution pH and are herein inferred as the prevalent adsorption mechanism throughout the entire solution pH range. Similar results were obtained by Clark et al. [13] and Alves et al. [17] when studying the adsorption mechanism of this amino acid using adsorbent produced from defective coffee beans and corn cobs, respectively. Based on these results (Figure 5), the charcoal dosage of 10 g L^−1^, pH = 4.0 and temperature of 25 °C were the most efficient initial parameters obtained under experimental conditions, and thus, selected to be used in subsequent experiments.

### 3.4. Effect of Initial Phenylalanine Concentration and Contact Time

**Figure 6:**
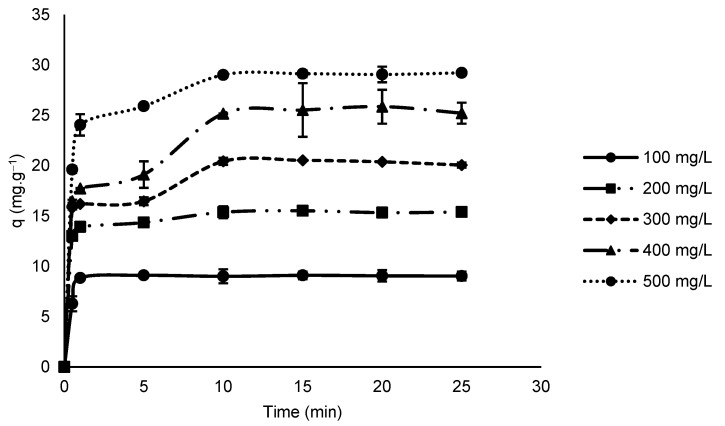
Effect of variations in the initial concentration of phenylalanine (25 °C, adsorbent dosage 10 g L^−1^ and pH 4).

An increase in the initial concentration of phenylalanine from 100 mg L^−1^ to 500 mg L^−1^ resulted in an adsorption capacity approximately three-times higher (Figure 6).

The observed increase was attributed to an increase in the driving force (concentration gradient of phenylalanine). When comparing this behavior with the result obtained by varying the adsorbent dosage, it can be observed that, in both cases, the increase in the adsorbate/adsorbent ratio positively influences the adsorption capacity of the adsorbent used. Clark et al. [13] and Alves et al. [17] also reported higher adsorption capacities of adsorbents produced from agro-industrial residues when there was an increase in initial phenylalanine concentration. 

The adsorption kinetic behavior showed two distinct adsorption steps. A fast initial adsorption step, where a 30 s contact resulted in the removal of 62.7 mg of phenylalanine per gram of adsorbent for a solution with initial concentration of 100 mg L^−1^, and 196 mg for a solution with initial concentration of 500 mg L^−1^. The second stage was of a slow adsorption, where a contact time of 10 min resulted in the removal of 27.3 mg of phenylalanine per gram of adsorbent for a solution with an initial concentration of 100 mg L^−1^, and 94.01 mg for a solution with initial concentration of 500 mg L^−1^. At higher temperatures, the same qualitative behavior of the adsorption process was observed. A contact time of 10 min was enough to ensure that equilibrium conditions were reached for all initial concentrations of phenylalanine evaluated (Figure 6). The rapid adsorption of phenylalanine by the AC produced in this work represents a significant improvement in the adsorption capability when compared to adsorbents prepared from corn cobs [17] and defective coffee beans [12], for which the contact times to reach equilibrium were 180 and 240 min, respectively.

Alves et al. [17] concluded that this difference could be explained by the mesoporous texture of its adsorbent being greater than that of the adsorbent produced by Clark et al. [13]. The data obtained in this work corroborate the explanation mentioned above, since the adsorbent herein prepared presented a mesoporous structure twice as large as the adsorbent obtained from corn cobs [17], and six-times larger than the adsorbent prepared from defective coffee beans [13]. In addition, the adsorbent produced in this work presented a smaller microporous structure when compared to the same adsorbents mentioned earlier.

### 3.5. Adsorption Equilibrium

The adsorption equilibrium data of L-phenylalanine at temperatures of 25, 35 and 45 °C were analyzed and adjusted by the two-parameter isotherm models of Langmuir and Freundlich [45], Temkim [46], Dubinin-Radushkevich [41] and by the three-parameter model of Langmuir–Freundlich [47]. The models’ equations and parameters used are listed in Table 5.

The best isotherm model fit was based on the highest coefficient of determination R^2^ [48] and the lowest chi-square coefficient χ^2^ [49]. The Freundlich model presented the best fit for all temperatures, suggesting that the adsorption process occurred on an energetically heterogeneous surface, which corroborates the data obtained by the IR spectra, and with the quantification of functional groups performed by the Boehm method that presented a variety of functional groups that interact differently with the adsorbate molecules. The values of the parameters K_LF_ (adsorption affinity constant) and n (index of heterogeneity) obtained from the Langmuir–Freundlich isotherm model used in this work, confirmed the best fit for the Freundlich isotherm model at the temperatures studied, since those parameters indicate a better approximation to the Freundlich model when they present low values.

**Figure 7:**
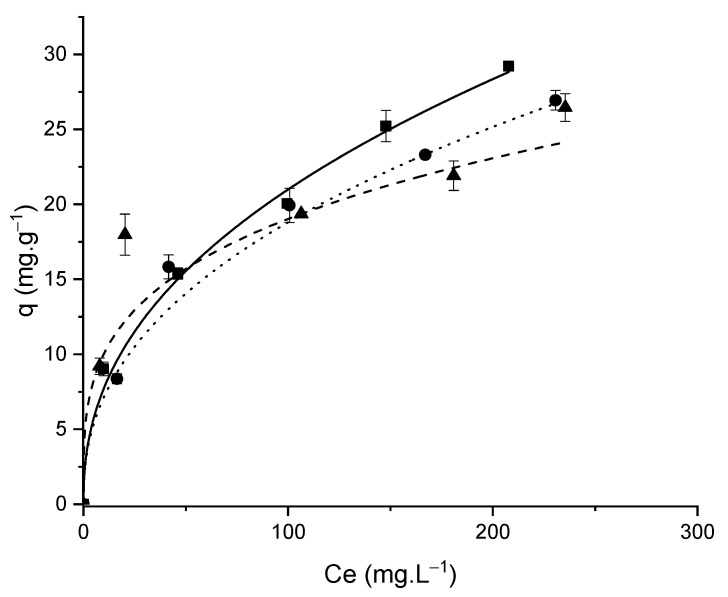
Adsorption isotherms (■ 25 °C; ● 35 °C; ▲ 45 °C—solid lines represent the fit of Freundlich model).

The favorable characteristic of the adsorptive process is indicated by the forms of the isotherm curves in the equilibrium adsorption capacity curves (Figure 7), and the values of the parameter n (characteristic of the Freundlich model) between 1 and 10 (Table 5) corroborate the graphical indication of favorability of this process at all temperatures.

Another important parameter referring to the Langmuir model is the separation factor or equilibrium parameter (RL), which determines whether an adsorption process is favorable or unfavorable [50], and is calculated by equation 5.

R_L_ = 1/(1 + K_L_C_O_) (5)

where K_L_ and C_O_ are the Langmuir constant and the initial concentration of L-phenylalanine, respectively. Adsorption is favorable when R_L_ < 1, irreversible adsorption occurs when R_L_ approaches zero, linear adsorption occurs when R_L_ = 1, and unfavorable adsorption occurs when R_L_ > 1 [51].

According to the R_L_ values presented in Table 5, the adsorption of L-phenylalanine is favorable at all temperatures studied, corroborating the data presented in Figure 7 and the values of the parameters from the Freundlich model.

An increase in temperature caused a decrease in the adsorbed amount, indicating an exothermic adsorption process, which was also confirmed by the decrease in adsorption heat (RT/b constant), and by the positive value of the constant b that is linked to the nature of the adsorption. Both parameters were obtained from Temkin’s isotherm model (Table 5). Similar results regarding the exothermic character of the phenylalanine adsorption process were also found in adsorbent produced from defective coffee beans and corn cobs [13,17]. The change in this adsorptive behavior with temperature can be explained by the greater strength of hydrophobic interactions between the molecules of the adsorbate at higher temperatures [13,52], which will consequently lead fewer molecules to leave the solution to be adsorbed onto the surface of the adsorbent.

The average energy (E) of adsorption per mole of adsorbate when transferred from the solution to the surface of the adsorbent can be obtained by fitting the Dubinin–Radushkevich isotherm model. Values between 8 and 16 kJ mol^−1^ indicate a chemical adsorption process [41] and, in this work, E values were measured between 10 and 15 kJ mol^−1^ suggesting chemisorption as a possible mode of adsorption.

### 3.6. Adsorption Kinetics

The removal rate of phenylalanine was investigated to help understand the adsorption mechanism and the pseudo-first order, pseudo-second order and intra-particle diffusion kinetic models used in this study were [53]:

ln(q_e_ − q_t_) = ln q_e_ − k_1_t (6)

1/q_t_ = 1/(k_2_q_e_^2^) + t/q_e_ (7)

q_t_ = K_p_ t^(1⁄2)^ + C (8)

where q_e_ (mg g^−1^) and q_t_ (mg g^−1^) are the adsorbed amounts of phenylalanine at equilibrium and at time t (min), K_1_ (min^−1^) is the adsorption rate constant of the pseudo-first order model, K_2_ (g mg^−1^ min^−1^) is the adsorption rate constant of the pseudo-second order model, K_p_ (mg g^−1^ min^−1/2^) is the intra-particle diffusion coefficient, and C (mg g^−1^) is the constant representing the boundary layer thickness. The representative model for adsorption kinetics was selected based on its ability to predict experimental data in terms of the highest coefficient of determination R^2^ [48] and the lowest chi-squared coefficient χ^2^ [49].

From the results in Table 6, it can be observed that the pseudo-second order model described the experimental data well, as it presented the highest R^2^ values, the lowest χ^2^ values, and good agreement between the experimental and calculated adsorption capacities for all the temperatures and concentrations studied. This result also indicates the chemical nature of the adsorption process involving valence forces through sharing or donating electrons between the adsorbate and the adsorbent [49], corroborating the data from the Dubinin–Radushkevich Isotherm model, which also indicated chemical adsorption. The pseudo-second order model was also the most adequate for describing the adsorption process of phenylalanine using adsorbents prepared from defective coffee beans [13], corn cobs [17] and date stones [18].

**Figure 8:**
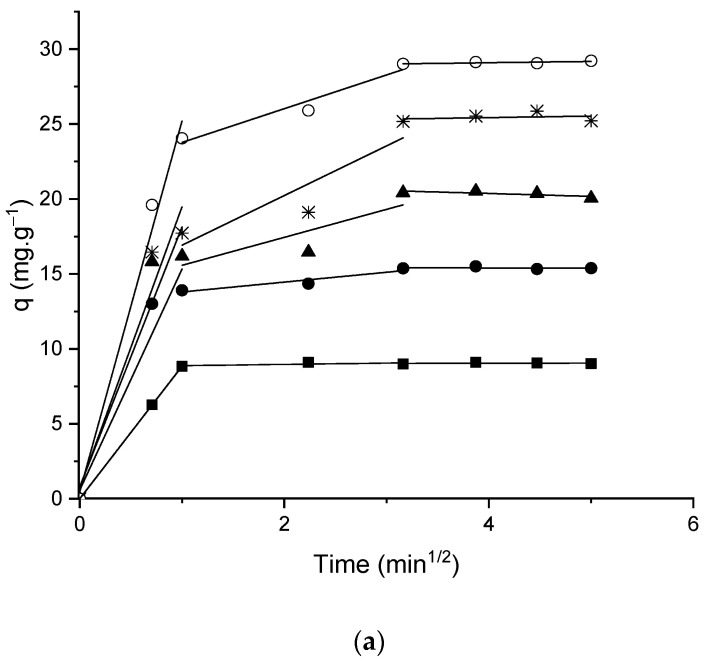

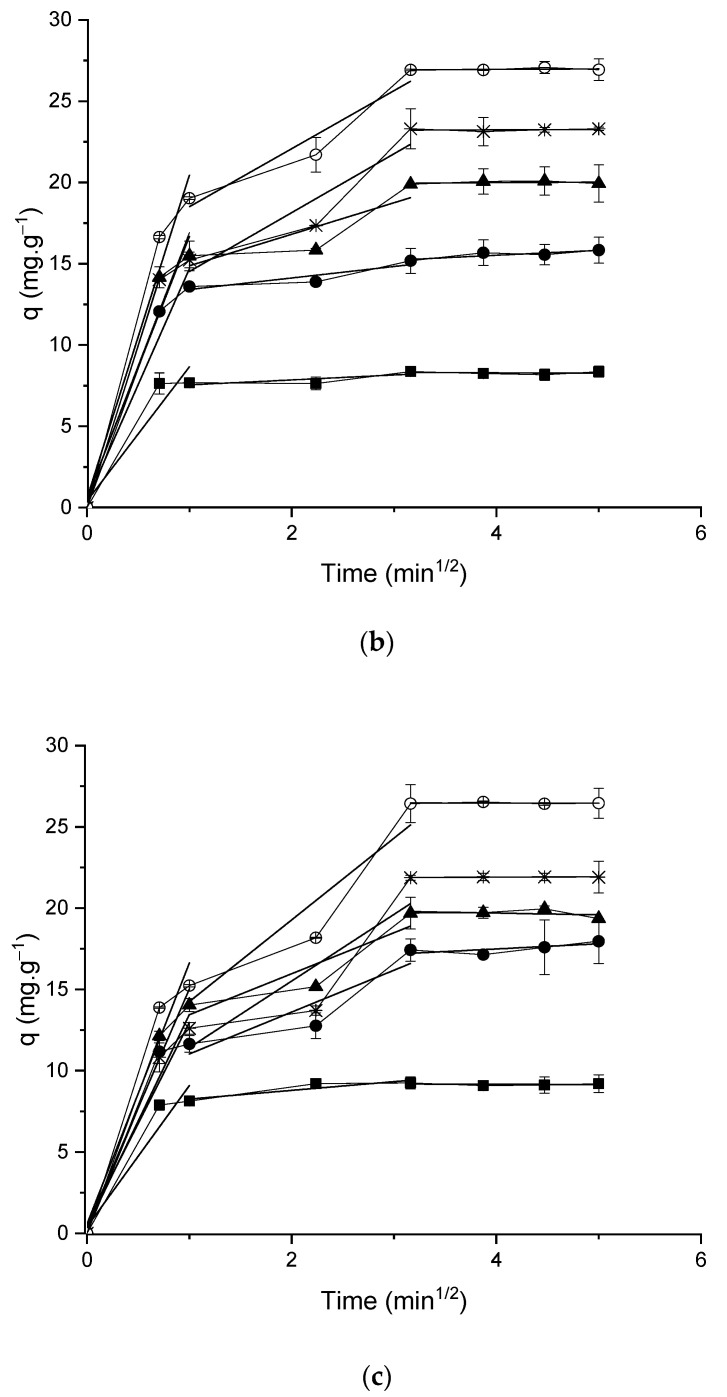
Intra-particle diffusion model for phenylalanine adsorption at (**a**) 25 °C, (**b**) 35 °C, (**c**) 45 °C (adsorbent dosage 10 g L^−1^; pH 4; initial concentration: ■ 100 mg L^−1^; ● 200 mg L^−1^; ▲ 300 mg L^−1^; ∗ 400 mg L^−1^; ◯ 500 mg L^−1^).

The intra-particle diffusion model was used to explain the diffusion mechanism and the steps of adsorption kinetics. According to the data in Table 6 and the curves in Figure 8, intra-particle diffusion in this study was the control step only for the initial concentration of phenylalanine of 100 mg L^−1^ and temperature of 25 °C. For the other concentrations and temperatures, the diffusion through the film is also an important step as the C values are different from zero and resulted in a q_t_ × t^(1⁄2)^ curve that does not pass through the origin [54].

The behavior reported for the temperature of 25 °C, where the adsorption mechanism changed with increasing concentration, can be explained by the greater possibility of hydrophobic bond formation between phenylalanine molecules in solution at the highest concentrations, which would hinder the binding of these molecules with the adsorbent.

The exothermic character of the adsorption process proved by the data obtained from the Temkin isotherm model (Table 5 and Figure 7), may explain the change in the adsorption control mechanism with the increase in temperature for the concentration of 100 mg L^−1^, since at higher temperatures the hydrophobic interactions between the molecules of the adsorbate have greater strength [13,52].

The identification of three distinct adjusted lines for all concentrations and temperatures studied (Figure 8) refer to diffusion in mesopores, micropores, and equilibrium, respectively. The increase in slope values for the first two lines with the increase in the initial concentration from 100 to 300 mg L^−1^ of phenylalanine may have occurred due to an increase in the mass transfer driving force between the solution and the adsorbent [13,17]. These results suggest diffusion as the control mechanism in micro- and mesopores.

### 3.7. Thermodynamic Adsorption Characterization

The adsorption mechanism may occur via physical or chemical processes, and the evaluation of thermodynamic parameters may suggest the presence or predominance of one of them. The thermodynamic parameters of adsorption presented in this work were calculated by [55,56]

K_C_ = q_e_/C_e_ (9)

ΔG° = −RTln(ρK_C_) (10)

ln(ρK_C_) = ((ΔS^O^)/R) − ((ΔH°)/RT) (11)

E_a_ = Rln(K_C (313K)_)/K_C (298k)_)/((1/T_1_) − (1/T_2_)) (12)

where K_C_ is the equilibrium constant; q_e_ and C_e_ are, respectively, the equilibrium concentration of phenylalanine in the solid phase (mg/L) and in solution (mg/L); ΔG° is the Gibbs energy variation (KJ mol^−1^), R is the universal gas constant (8.314 J mol^−1^ k^−1^), T is the absolute temperature in K and ρ is the specific mass of water (g L^−1^). While K_C_ and ΔG° are directly determined by their equations, ΔH° (enthalpy change) and ΔS° (entropy change) were calculated from the slope and intercept of the ln(ρKc)× 1/T curve. The activation energy (E_a_ KJ mol^−1^) was obtained directly from equation 12. The calculated thermodynamic data are presented in Table 7.

The value of ΔG° < 0 suggests a favorable and spontaneous adsorption process at all studied temperatures. This result agrees with the values of parameter n (characteristic of the Freundlich model) between 1 and 10 (Table 5), and the favorable curve indication of this process which was observed for all temperatures (Figure 7).

The magnitude of ΔH° between 2.1 and 20.9 kJ mol^−1^ (Table 7) suggests a physical adsorption process (Liu, 2009). The negative value obtained indicating an exothermic process, which was confirmed by a decrease in adsorption capacity (Table 5 and Table 6, Figure 7), decrease in the equilibrium constant (Table 7) with increasing temperature, decrease in the heat of adsorption, and by the positive value of the constant linked to the nature of the adsorption (both parameters of the Temkim isotherm—Table 5). The value of ΔS° > 0 indicates that the organization of phenylalanine molecules at the solid/solution interface becomes more random in the adsorption process.

The E_a_ < 0 (Table 7) indicates a physical adsorption process, since its magnitude is lower than 42 kJ mol^−1^ [57], with the negative value confirming the decrease in the amount of phenylalanine adsorbed with increasing temperature, thus, corroborating the exothermic character of the adsorption [58,59]. Considering these results, it may be safely inferred that physical and chemical adsorption may occur simultaneously during the adsorption process, since the kinetic and equilibrium results indicated the occurrence of chemical processes, and the thermodynamic parameters indicated the occurrence of physical processes. Physical adsorption was also inferred for adsorption of phenylalanine on adsorbents prepared from palm seed [18].

### 3.8. Adsorption Mechanism

**Figure 9:**
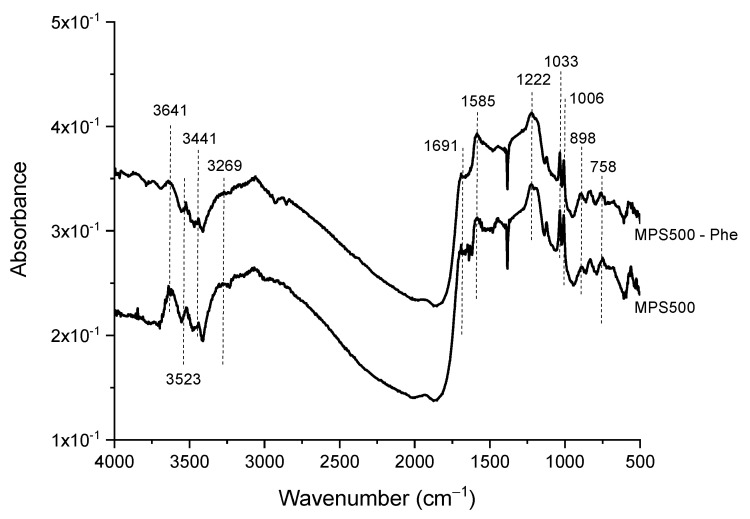
FTIR spectra of the prepared adsorbent before (MPS500) and after adsorption (MPS500−Phe).

The functional groups involved in the adsorption mechanism were analyzed by FTIR. The FTIR spectra of the adsorbent before and after the adsorption of phenylalanine are presented in Figure 9.

The changes in band intensities (Figure 9) lead us to infer that dipole–dipole hydrogen bonds, Yoshida hydrogen bonds, n–π interactions and nonpolar interactions of the functional groups present on the surface of MPS500 and the phenylalanine molecule may be involved in the adsorption mechanism.

In addition to the change in the bands’ intensities, band displacements at 3641, 3523, 3441 and 3269 cm^−1^ were also observed. The first band is related to the stretching of the –OH group in phenols, and the other bands are related to the stretching of the –OH group in phenols associated with intermolecular hydrogen bond. The presence of these groups at the adsorbent surface is confirmed by the amount of hydroxyl groups determined by the Boehm method (5.54 mmol g^−1^). The data obtained by the Boehm method and FTIR lead us to suggest that two types of hydrogen bond interactions may be occurring between the adsorbent MPS500 and phenylalanine. The first interaction known as a dipole–dipole hydrogen bond occurs between the hydrogen donor hydroxyl groups on the surface of MPS500, and hydrogen acceptor atoms on phenylalanine (such as nitrogen and oxygen). The second interaction known as the Yoshida hydrogen bond between the hydroxyl groups on the surface of MPS500 and the aromatic rings of phenylalanine [60]. Band shift related to –OH group stretching was also observed after adsorption of tryptophan on activated carbon produced from date stones [33], and on adsorption of methylene green on commercial activated carbon [56].

The displacement of the bands in 1222 cm^−1^ (stretch vibrations of the C–O bond present in acids, alcohols, phenols, esters and ethers), 1006 cm^−1^ (S=O bond stretching vibrations), 898 cm^−1^ (P–O–P bond stretch vibration) and 758 cm^−1^ (P–C bond stretching vibration), and the increase in intensity of bands 1691 cm^−1^ (C=O bond stretching vibrations in carboxylic acid and lactone) and 1033 cm^−1^ (S=O bond stretching vibrations) suggest possible n–π interactions between these groups and the phenylalanine molecule. The n–π interactions or interactions between electron donors and acceptors [61] can occur between oxygen-containing groups, acting as electron donors on the surface of MPS500, and the aromatic rings of phenylalanine, acting as electron acceptors. Therefore, it can be inferred that these interactions increased the intensity or promoted the displacement of those bands after the contact of phenylalanine with the adsorbent. A similar behavior was shown in the adsorption of tryptophan on date-seed carbon activated with phosphoric acid [56].

The increase in intensity of the 1585 cm^−1^ band referring to the C=C bond of aromatic compounds, allows us to infer the existence of a π–π interaction between the adsorbent surface and the phenylalanine molecule. Alves et al. [17] also observed π–π interactions after phenylalanine adsorption using active carbon prepared from corn cobs. The MPS500 prepared in this study presented an adsorption mechanism that quickly reaches equilibrium (Figure 6 and Figure 7), and this behavior can be explained by the synergism of two factors: the number of mesopores facilitating diffusion of the adsorbate molecule within the adsorbent; and the diversity of chemical functional groups on the surface of the adsorbent that can establish dipole–dipole hydrogen bonds, Yoshida hydrogen bonds, n–π interactions and nonpolar interactions with the phenylalanine molecule.

### 3.9. Adsorption in Multicomponent Solutions

The adsorption equilibrium study was conducted for the binary system phenylalanine—tyrosine and the adsorption dynamics was evaluated by the q_e_’/q_e_ ratio, where q_e_’ is the adsorption capacity of the primary component in a binary system and q_e_ the adsorption capacity of the primary component in a unitary or monocomponent system, both obtained experimentally. There are three possible behaviors occuring: synergism (q_e_’/q_e_ > 1, where the effect of the mixture is greater than that of the individual adsorbates), antagonism (q_e_’/q_e_ < 1, indicating that the effect of the mixture is smaller than that of each of the individual adsorbates), and there is no interaction (q_e_’/q_e_ = 1), inferring that the mixture has no effect on the adsorption of each component in the mixture [62].

**Figure 10:**
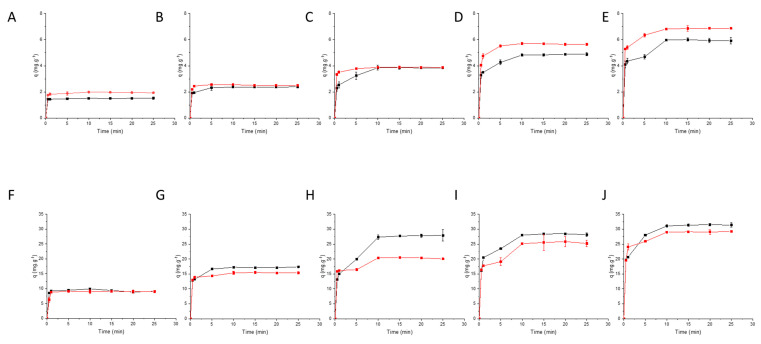
Dynamics of adsorption capacity in the phenylalanine–tyrosine binary system: (**A**–**E**) (●—monocomponent solution of Tyr 50 mg L^−1^ e ● multicomponent solution of Tyr (50 mg L^−1^)—Phe (**A**) 100 mg L^−1^; (**B**) 200 mg L^−1^; (**C**) 300 mg L^−1^; (**D**) 400 mg L^−1^; (**E**) 500 mg L^−1^) e (**F**–**J**) (●—monocomponent solution of Phe 500 mg L^−1^ e ● multicomponent solution of Phe (500 mg L^−1^)—Tyr (**F**) 20 mg L^−1^; (**G**) 30 mg L^−1^; (**H**) 50 mg L^−1^; (**I**) 75 mg L^−1^; (**J**) 100 mg L^−1^).

In the binary system, antagonism occurred where tyrosine was the primary amino acid antagonism, i.e., the process of tyrosine adsorption in the presence of the interferent phenylalanine was compromised, and there was a decrease in tyrosine adsorption (Figure 10A–E).

In the binary system, synergism was observed when phenylalanine was the primary amino acid and tyrosine was the interferent, that is, in the process of phenylalanine adsorption with the presence of interferent there was an increase in adsorption of phenylalanine (Figure 10F–J). These data reveal an important advantage in the charcoal used, since it was more selective for phenylalanine adsorption and with less adsorption of tyrosine in a competitive system.

### 3.10. Comparison with Literature

The MPS 500 carbon obtained in this study showed a maximum adsorption capacity that varied between approximately 1.5- and 150-times greater when compared to other adsorbents in the literature (Table 8), such as mesoporous materials [63], organic–inorganic hybrid membranes [64] and macroporous resins [65], and with the advantage of using a 20-fold lower L-phenylalanine concentration when compared to the adsorption conditions used for mesoporous materials and macroporous resins. Furthermore, the adsorbent dosage was 20-times lower than that used for macropourous resin and 1.6-times lower for organic–inorganic hybrid membranes.

## 4. Conclusions

The adsorption of L-phenylalanine using sunflower meal as precursor material for the production of activated carbon was investigated in this work. The adapted methodology to functionalize the adsorbent with sulfonic groups demonstrated advantages in terms of execution time, energy expenditure, the amount of reagents used, and the amount of adsorbed amino acid. Moreover, efficient changes were produced in chemical groups, thermal stability and textural properties. The optimization parameters indicated a higher adsorption capacity (39.64 mg.g^−1^) at pH = 4; temperature of 25 °C and adsorbent dosage of 10 g L^−1^. The adsorption process occurred on an energetically heterogeneous surface since the Freundlich isotherm model was characterized as the best fit and this result corroborates the diversity of chemical groups found in the FTIR spectra and in the Boehm methodology. The pseudo-second order model fitted well the experimental data, indicating a chemical nature in the adsorption process and that corroborated with the data found in the Dubinin–Radushkevich isotherm model. The thermodynamic parameters indicate a spontaneous (ΔGO < 0) and exothermic (ΔHO < 0) process with greater randomness of the system (ΔSO > 0) and physical adsorption. The adsorption mechanism can be explained by the synergism of textural properties and chemical interactions such as dipole–dipole hydrogen bonding, Yoshida hydrogen bonds, n–π interactions and nonpolar interactions. The charcoal produced had the advantage of being selective for phenylalanine in a competitive system with tyrosine. This work contributes to the understanding of L-phenylalanine adsorption on surfaces functionalized with sulfonic groups. With this work as a guide, future studies can be conducted for other chemical modifications on the surfaces of adsorbents, in order to elucidate the adsorption mechanism of this amino acid or other adsorbates bearing similar chemical structures.

## Figures and Tables

**Table 1:** Gravimetric carbonization yield, adsorption capacity (q_t_), performance index, and total removal efficiency (R%) of charcoal samples.

Sample	Yield (%)	$q_{t}\;(\mathrm{mg}\;g^{-1}\;\mathrm{of}\;\mathrm{AC})$	$I\;(\mathrm{mg}\;g^{-1}\;\mathrm{of}\;\mathrm{Meal})$	R (%)
M500	30.23 ± 1.84 ^c^	7.28 ± 1.83 ^c^	2.20 ± 0.57 ^c^	12.79 ± 3.22 ^c^
MP500	49.01 ± 3.99 ^a^	17.50 ± 0.09 ^b^	8.58 ± 0.70 ^b^	33.62 ± 0.18 ^b^
MPS500	40.46 ± 3.29 ^b^	25.75 ± 0.67 ^a^	10.42 ± 0.89 ^a^	49.02 ± 1.40 ^a^

The gravimetric yield, adsorption capacity, performance index and total removal efficiency (R%) values correspond to the averages and their respective standard deviations. Means with different letters in the same column indicate that the values are significantly different from each other as determined via the Tukey test (*p* < 0.05).

**Table 2:** Textural properties of prepared adsorbents compared to other adsorbents from agro-industrial waste.

Adsorbent	S_T_ (m^2^ g^−1^)	V_T_ (cm^3^ g^−1^)	Mesopores				Micropores			
			S_me_		V_me_		S_mi_		V_mi_	
			(m^2^ g^−1^)	(%)	(cm^3^ g^−1^)	(%)	(m^2^ g^−1^)	(%)	(cm^3^ g^−1^)	(%)
M500 ^1^	1.26	1.52 × 10^−3^	1.14	90.43	1.41 × 10^−3^	92.53	0.12	9.56	1.13 × 10^−4^	7.44
MP500 ^1^	155.32	2.57 × 10^−1^	93.39	60.13	2.24 × 10^−1^	87.03	61.80	39.79	3.23 × 10^−2^	12.58
MPS500 ^1^	317.31	3.82 × 10^−1^	128.29	40.43	2.92 × 10^−1^	76.51	188.86	59.52	8.85 × 10^−2^	23.16
CCAC ^2^	893.70	6.12 × 10^−1^	192.40	21.53	2.48 × 10^−1^	40.52	610.60	68.32	2.88 × 10^−1^	47.06
ADC ^3^	490.80	2.77 × 10^−1^	31.00	6.32	3.40 × 10^−2^	12.27	421.49	85.88	2.00 × 10^−1^	72.02
A–H–2-500 ^4^	727.14	4.03 × 10^−1^	-	-	1.29 × 10^−1^	32.01	-	-	2.74 × 10^−1^	67.89

^1^ This study; ^2^ [17]; ^3^ [13], ^4^ [27].

**Table 3:** Adsorption capacity (q_e_) of phenylalanine in activated carbon produced from agro-industrial by-products.

AC Precursor Material	q_e_ (mg g^−1^)	Contact Time (min)	Reference
Corn cobs	32.92	720	[17]
Defective coffee beans	28.74	360	[13]
Sunflower meal	25.75	25	This work

All experiments were performed at 25 °C, initial phenylalanine concentration of 500 mg L^−1^ and adsorbent concentration of 10 g L^−1^.

**Table 4:** EDS elemental composition of M500, MP500 and MPS500 carbons.

Element	M500		MP500		MPS500	
	% M *	% A **	% M *	% A **	% M *	% A **
Carbon	63.28	74.19	57.59	71.59	73.11	79.71
Oxygen	22.87	20.13	18.24	17.02	22.34	18.29
Potassium	7.11	2.56	2.54	0.97	0	0
Phosphorus	3.57	1.62	20.36	9.81	1.23	0.52
Magnesium	1.48	0.86	0.20	0.13	0.06	0.03
Calcium	1.38	0.48	0.57	0.21	0	0
Aluminum	0.27	0.14	0.33	0.18	0.24	0.12
Sulfur	0.04	0.02	0.00	0.00	1.51	0.62
Sodium	0.00	0.00	0.00	0.00	0.07	0.04
Silicon	63.28	74.19	57.59	71.59	73.11	79.71

* % M = percentage by mass of the element and ** % A = percentage by number of atoms of the element.

**Table 5:** Adsorption isotherm models and respective fitness parameters.

Model	Equation	Parameter Values			R^2^			χ^2^		
		25 °C	35 °C	45 °C	25 °C	35 °C	45 °C	25 °C	35 °C	45 °C
Langmuir	$q_{e}=\frac{q_{m}K_{L}C_{e}}{1+K_{L}C_{e}}$	K_L_ = 0.0122	K_L_ = 0.0247	K_L_ = 0.0954	0.9925	0.9997	0.9999	44.56	3.57	13.89
		q_m_ = 39.62	q_m_ = 29.02	q_m_ = 21.27						
		R_L_ = 0.67	R_L_ = 0.58	R_L_ = 0.33						
Freundlich	$q_{e}=K_{F}\;C_{e}^{1/n}$	K_F_ = 2.853	K_F_ = 2.717	K_F_ = 5.290	0.9989	0.9997	1.0000	6.39	3.81	6.10
		n = 2.308	n = 2.381	n = 3.597						
Temkin	$q_{e}=(RT/b)\ln\left(K_{T}C_{e}\right)$	K_T_ = 0.2516	K_T_ = 0.2269	K_T_ = 1.3645	0.9919	0.9998	1.0000	48.52	2.15	8.21
		RT/b = 6.93	RT/b = 6.43	RT/b = 3.90						
		b = 0.3578	b = 0.3981	b = 0.6789						
Langmuir-Freundlich	$q_{e}=\frac{K_{LF}q_{m}C_{e}^{n}}{1+K_{LF}C_{e}^{n}}$	K_LF_ = 9.04 × 10^−5^	K_LF_ = 5.14 × 10^−6^	K_LF_ = 8.51 × 10^−6^	0.9925	0.9997	0.9999	59.42	4.75	18.52
		q_m_ = 39.64	q_m_ = 29.02	q_m_ = 21.27						
		n = 7.40 × 10^−3^	n = 2.08 × 10^−4^	n = 8.92 × 10^−5^						
Dubinin-Radushkevich (D-R)	q_e_ = q_m_ exp(−Be^2^)	E = 10.38	E = 11.14	E = 14.56	0.9741	0.9939	0.9311	13.62	3.06	7.24
	e = RT ln(1 + 1/C_e_)	q_m_ = 101.93	q_m_ = 82.19	q_m_ = 47.22						
	$E=1/\sqrt{2B}$									

q_e_ (mg g^−1^) is the equilibrium adsorption capacity; C_e_ (mg L^−1^) is the solute concentration in the aqueous solution, after equilibrium; q_m_ (mg g^−1^) is the maximum adsorption capacity; R is the universal gas constant (8.314 × 10^−3^ KJ mol^−1^ k^−1^); T(K) is the absolute temperature; E (kJ mol^−1^) is the mean free energy; the ramaining constants are empirical parameters associated to each specific model.

**Table 6:** Kinetic parameters for phenylalanine adsorption.

	Phe Initial Concentration (mg L^−1^)				
	100	200	300	400	500
25 °C					
q_e_ (experimental)	9.02	15.39	20.06	25.22	29.22
Pseudo-first-order					
k_1_ (min^−1^)	2.528	3.747	2.930	1.848	2.184
q_e_ (estimated) (mg g^−1^)	9.10	15.07	19.32	24.00	28.37
R^2^	0.9959	0.9914	0.9461	0.9202	0.9856
χ^2^	0.049	0.281	2.979	7.103	1.675
Pseudo-second-order					
k_2_ (g mg^−1^min^−1^)	2.058	0.565	0.172	0.076	0.136
q_e_ (estimated) (mg g^−1^)	9.07	15.46	20.48	26.00	29.47
R^2^	0.9999	0.9998	0.9981	0.9964	0.9996
χ^2^	0.0001	0.0001	0.0005	0.0006	0.0001
Intra-particle-difusion					
k_P_ (mg g^−1^min^−1/2^)	8.84	14.74	17.33	18.75	24.73
C	0.00	0.59	0.81	0.72	0.48
R^2^	0.9999	0.9476	0.9293	0.9505	0.9870
35 °C					
q_e_ (experimental)	8.36	15.83	19.93	23.30	26.93
Pseudo-first-order					
k_1_ (min^−1^)	5.548	2.980	2.343	1,582,000	1,714,000
q_e_ (estimated) (mg g^−1^)	8.09	15.13	19.00	21.94	25.77
R^2^	0.9905	0.9835	0.9446	0.9194	0.9507
χ^2^	0.090	0.540	2.977	6.120	4.975
Pseudo-second-order					
k_2_ (g mg^−1^min^−1^)	1.006	0.238	0.128	0.074	0.085
q_e_ (estimated) (mg g^−1^)	8.33	15.87	20.32	23.83	27.48
R^2^	0.9996	0.9994	0.9980	0.9966	0.9983
χ^2^	0.0006	0.0003	0.0005	0.0006	0.0002
Intra-particle-difusion					
k_P_ (mg g^−1^min^−1/2^)	8.25	14.24	16.32	16.07	19.86
C	0.41	0.45	0.59	0.60	0.59
R^2^	0.9212	0.9657	0.9556	0.9553	0.9702
45 °C					
q_e_ (experimental)	9.21	17.97	19.36	21.91	26.46
Pseudo-first-order					
k_1_ (min^−1^)	3.280	1.741	1.761	1.170	1.213
q_e_ (estimated) (mg g^−1^)	9.07	16.45	18.69	20.26	24.75
R^2^	0.9908	0.9029	0.9379	0.8667	0.8947
χ^2^	0.1075	4.1496	3.3344	9.7289	10.9570
Pseudo-second-order					
k_2_ (g mg^−1^min^−1^)	2.091	0.082	0.114	0.047	0.048
q_e_ (estimated) (mg g^−1^)	9.19	18.20	19.98	22.79	27.33
R^2^	0.9999	0.9954	0.9969	0.9891	0.9938
χ^2^	0.0001	0.001	0.0008	0.002	0.001
Intra-particle-difusion					
k_P_ (mg g^−1^min^−1/2^)	8.69	12.42	14.62	13.11	16.05
C	0.39	0.54	0.41	0.35	0.57
R^2^	0.9326	0.9374	0.9732	0.9753	0.9570

**Table 7:** Thermodynamic parameters of phenylalanine adsorption.

T(K)	Van’t Hoff Equation	K_C_	ΔG° (KJ mol^−1^)	ΔH° (KJ mol^−1^)	ΔS° (KJ mol^−1^)	E_a_ (KJ mol^−1^)
298	y = 1627.5x + 1.9556 R^2^ = 0.9935	1.71	−18.45	−13.53	0.02	−13.50
308		1.40	−18.55			
318		1.21	−18.78			

**Table 8:** Comparison of the maximum adsorption capacity (Q_m_) of L-phenylalanine, properties textural and adsorption conditions for various adsorbents.

Adsorbent	Q_m_ (mg g^−1^)	Textural Properties			Adsorption Conditions				Reference
		S_T_ (m^2^ g^−1^)	V_T_ (cm^3^ g^−1^)	Average Pore Diameter (nm)	Phe Concentration (mg L^−1^)	pH	T (°C)	Dosage Adsorbent (g L^−1^)	
Mesoporous materials C_SBA-15_, C_SBA-16_ and C_KIT-6_	0.27–0.31	1074–1210	0.97–1.02	3.29–4.13	80–11,600	5.6	20	5	[63]
Organic–inorganic hybrid membranes	1.21	-	-	-	41–111	-	25	16	[64]
Macroporous resins NKA-9, HD-2, HP-20, NKA-2 e S-8	12.80–26.90	40–600	-	-	10,000	6.8	25	100	[65]
Mesoporous silica SBA-3	37.33	1233	0.78	2.53	80–11,600	5.6	20	5	[66]
Activated meal sunflower	39.64	317.31	0.38	4.78	100–500	4	25	10	This study
Calcined CuZnAl-CO_3_ layered double hydroxides	46.4	-	-	-	0–25	6.7	40–80	1	[67]
Activated defective coffee beans	69.45	490.80	0,28	2	300–1500	6	25	10	[13]
Activated corn cobs	109.20	893.70	0.61	1.30–3.60	200–1500	6	25	10	[17]
Activated date stones	133.33–188.32	1209–1235	0.55–0.63	1.48–1.59	50–1000	5.7	20	1	[18]

S_T_, total surface area; V_T_, total pore volume.

## Data Availability

All data generated and analyzed in this study are included in the manuscript.
